# Integrating Automated Labeling Framework for Enhancing Deep Learning Models to Count Corn Plants Using UAS Imagery

**DOI:** 10.3390/s24196467

**Published:** 2024-10-07

**Authors:** Sushma Katari, Sandeep Venkatesh, Christopher Stewart, Sami Khanal

**Affiliations:** 1 Department of Food, Agricultural, and Biological Engineering, Ohio State University, 590 Woody Hayes Dr, Columbus, OH 43210, USA; 2 Google, Kirkland, WA 98033, USA; kvsandy22@gmail.com; 3 Department of Computer Science and Engineering, Ohio State University, 590 Woody Hayes Dr, Columbus, OH 43210, USA; stewart.962@osu.edu

**Keywords:** UAS, plant stand count, crop rows, automatic labeling

## Abstract

Plant counting is a critical aspect of crop management, providing farmers with valuable insights into seed germination success and within-field variation in crop population density, both of which are key indicators of crop yield and quality. Recent advancements in Unmanned Aerial System (UAS) technology, coupled with deep learning techniques, have facilitated the development of automated plant counting methods. Various computer vision models based on UAS images are available for detecting and classifying crop plants. However, their accuracy relies largely on the availability of substantial manually labeled training datasets. The objective of this study was to develop a robust corn counting model by developing and integrating an automatic image annotation framework. This study used high-spatial-resolution images collected with a DJI Mavic Pro 2 at the V2–V4 growth stage of corn plants from a field in Wooster, Ohio. The automated image annotation process involved extracting corn rows and applying image enhancement techniques to automatically annotate images as either corn or non-corn, resulting in 80% accuracy in identifying corn plants. The accuracy of corn stand identification was further improved by training four deep learning (DL) models, including InceptionV3, VGG16, VGG19, and Vision Transformer (ViT), with annotated images across various datasets. Notably, VGG16 outperformed the other three models, achieving an F1 score of 0.955. When the corn counts were compared to ground truth data across five test regions, VGG achieved an R^2^ of 0.94 and an RMSE of 9.95. The integration of an automated image annotation process into the training of the DL models provided notable benefits in terms of model scaling and consistency. The developed framework can efficiently manage large-scale data generation, streamlining the process for the rapid development and deployment of corn counting DL models.

## 1. Introduction

Plant counting is a critical task of crop scouting [1], offering farmers pivotal insights into seed germination success rates, within-field variation in crop population density [2,3], and traits related to crop quality and crop yield [4]. It can also be used as a parameter for understanding the effects of different crop management practices on crop yield and nutrient quality. A study found that varying plant densities influence forage corn’s nutritive quality, with higher densities leading to reduced forage quality [5]. Conversely, different tillage practices were found to have no notable effect on forage quality.

The conventional approach of plant counting relies on manual scouting, which is time and labor intensive, and incurs high costs. Recent advancements in remote sensing and machine learning (ML) techniques have offered alternatives by enabling the implementation of ML algorithms for plant counting without the need for on-site scouting. Images captured via Unmanned Aerial Systems (UASs) provide detailed information about crop fields [6,7], which is instrumental in estimating the pant stand counts through various techniques such as image enhancement, ML, and deep learning (DL) algorithms [8].

Image enhancement techniques employ computer vision approaches such as thresholding, edge detection, and contrast enhancement to map plant densities in crop fields. Donmez et al. (2021) [9] applied morphological operations such as erosion and dilation on high-resolution (3.7 cm) five-band UAS images to count citrus trees. They used the Connected Components Labeling (CCL) algorithm on morphologically processed images to automatically detect and count citrus trees in dense patches. While image enhancement techniques alone provided accurate plant count estimates, the use of ML or DL models can enhance accuracy further. Xia et al. (2019) [10] demonstrated this improvement using a Support Vector Machine (SVM) and Maximum Likelihood Classifier (MLC) for counting cotton plants, where the SVM outperformed the MLC. Similarly, Banerjee et al. (2021) [11] achieved a higher accuracy (R^2^ = 0.86) in estimating wheat seedling count using the Gaussian process regression (GPR) model on UAS-based multispectral images when compared to support vector regression and regression trees. Tavus et al. (2015) [12] combined image morphological and ML techniques on UAS images in estimating plant stand counts and achieved an accuracy of 87.7% using the K-Nearest Neighbor (K-NN) classification algorithm, indicating the potential of merging multiple techniques for precise plant stand count estimation.

Recent advancements in ML and deep-learning (DL) algorithms have significantly improved plant stand count estimation. Osco et al. (2021) [13] developed a Convolutional Neural Network (CNN) with a two-branch structure for locating crop plantation rows and counting plant stands, achieving around 90% accuracy across various crops. Zhang et al. (2020) [14] addressed challenges related to canopy overlap by integrating leaf count information into their CNN model, also achieving an accuracy of over 90% in estimating rapeseed plant count. Another study by Zhang et al. (2020) [15] evaluated the Scale Sequence Residual U-Net (SS Res U-Net) algorithm, finding it more accurate and computationally efficient than other U-Net variants. 

In contrast, Vong et al. (2021) [16] observed that U-Net’s performance in segmenting and counting plants varied with residue cover in UAS-based images, noting reduced accuracy with increased residue. Wang et al. (2021) [17] used YOLOv3 and a Kalman filter for counting seedlings amidst intricate soil backgrounds using images captured by a moving ground platform, achieving 98% accuracy during growth stages V2–V3. However, the use of ground-based machines can be time consuming and challenging to maneuver without disturbing crops.

The R-CNN family, including Mask R-CNN, has also been used for crop counting. Machefer et al. (2020) [18] refined the Mask R-CNN model for the precise counting of potato and lettuce crops, achieving an accuracy of 78% and 92%, respectively. Their research emphasized the superiority of fine-tuning the pre-trained Mask R-CNN compared to manually tuned computer vision algorithms. Given the computational expense of training R-CNN models, Lu and Cao (2020) [19] implemented faster TasselNetV2+ for counting wheat, maize, and sorghum plants and found that it outperformed Faster R-CNN in terms of computation time and performance. Additionally, a study [20] integrated YOLOv5 with a Convolutional Block Attention Module (CBAM) for rapeseed inflorescence counting, demonstrating superior performance with an R² of 0.96 compared to other DL models, including YOLOv4, TasselNetV2+, CenterNet, and Faster R-CNN. Despite the longer computation time required by these DL models in contrast to ML and image enhancement methods, they have been proven to be instrumental in achieving superior model performance.

While prior studies have developed various DL models for plant counting, the robustness of these models depends heavily on the quantity and quality of training data. Currently, the training data have been largely based on the manual annotation of images. Challenges such as limited access to ground truth data, high annotation costs, and uncertainties about data quality used for annotation can impede the implementation of state-of-the-art DL models [21,22]. 

Automating the image annotation process to create extensive high-quality training datasets ready for DL models holds tremendous opportunities for advancing predictive and prescriptive analytics in precision agriculture. However, only a few studies have explored semisupervised or semi-automated annotation for agricultural use cases including counting [23]. Some studies [24,25] have employed unsupervised domain adaptation (UDA) to enhance model performance utilizing unlabeled datasets from testing environments. Additionally, Shi et al. (2022) [26] proposed integrating a background-aware domain adaptation (BADA) module into existing DL networks to reduce background plant counting errors and improve model performance. 

Parallelly, studies have made progress in various computer vision techniques to improve object detection, including crop counting. Recent advancements include those of Bai et al. (2022) [27], who developed the peak detection algorithm for locating crop rows and plant seedlings of sunflower and maize plants using UAS-based RGB images. This is a swift and robust method, achieving an R^2^ of approximately 0.8 for both maize and sunflower. Similarly, Wu et al. (2019) [28] used thresholding, edge detection, and circular hough transformation algorithms to automatically delineate citrus tree boundaries from UAS multispectral images, demonstrating that around 80% of tree boundaries were accurately delineated and hence demonstrating the effectiveness of digital image processing techniques in plant counting. While these techniques have relatively lower accuracy compared to DL models, they require significantly less computation power. This presents an opportunity to combine computer vision methods, particularly image enhancement techniques, with DL models to improve crop counting accuracy without the need for manually labeled data as well as excessive computing resources. 

The objectives of this study are to (1) automate image annotation using image enhancement techniques and (2) evaluate the performance of state-of-the-art DL models, including CNN- and transformer-based architectures, trained on automatically annotated data for crop counting. This study aimed to streamline DL-based crop counting, improving accuracy while reducing the time needed to generate training data.

## 2. Materials and Methods

### 2.1. Study Area

**Figure 1:**
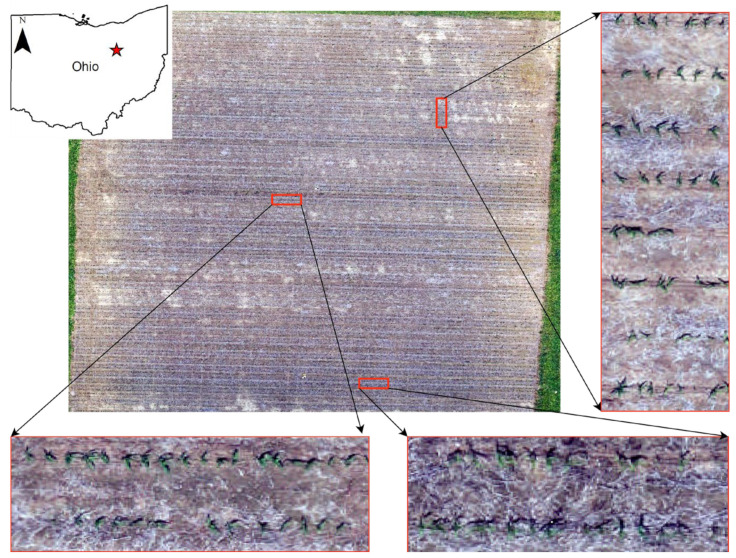
Study area—corn field located in Wooster, Ohio, USA.

This study was conducted based on images collected from a corn field located in the Wooster region, Ohio, USA (Figure 1). Corn seeds were sown in May 2019 with a plant population of 81,510 seeds/ha and were harvested around late October 2019.

### 2.2. Data Acquisition and Preprocessing

Images of the corn field were collected on 11 June 2019, using a DJI Mavic Pro 2 (Shenzhen, China) equipped with an RGB camera during the V2 to V4 growth stages. In general, corn growth stages are divided into Vegetative (V) and Reproductive (R) stages [29], with V(n) stages, where n represents the number of visible leaf collars. The V2–V4 stage, where corn plants develop 2–4 leaves leaf collars, was chosen because plant canopies typically do not overlap, making it easier to accurately identify corn plants in aerial images [16]. To avoid any potential variation in color across the collected images, UAS flights were conducted around noon with a clear sky at a height of 26 m. Additionally, in the RGB camera settings, the white balance was set to fixed values.

**Figure 2:**
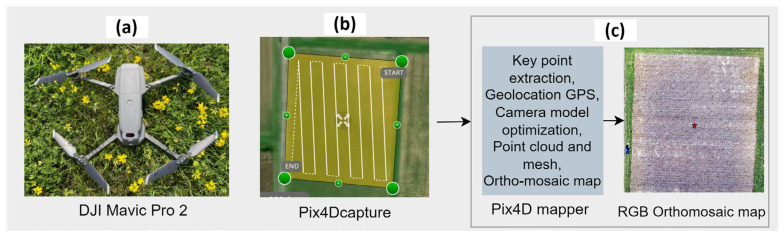
Data acquisition and image preprocessing steps: (**a**) UAS used for data collection, (**b**) flight path planning using the Pix4Dcapture software, and (**c**) steps involved in generating an ortho mosaic RGB map of an entire field.

Pix4Dcapture (4.4) software was used for flight planning with 85% frontal and 75% side overlap, and ground control points were established before UAS surveys to facilitate image geo-rectification. Individual images collected via UAS were stitched using the Pix4Dmapper (4.3) software and a georeferenced RGB image with a pixel resolution of 1.5 cm × 1.5 cm was created to provide a seamless representation of the entire corn field used in this study (Figure 2). 

### 2.3. Plant Counting Framework

**Figure 3:**
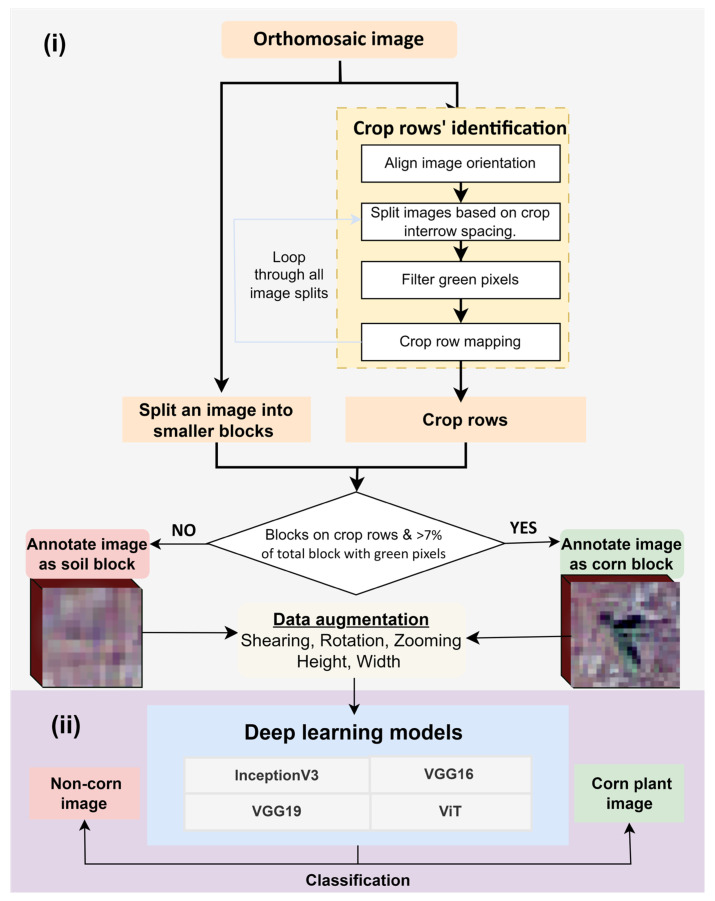
Automatic plant counting framework in two steps. (**i**) Step 1: Tasks for automatically annotating the UAS images for identifying corn plant and soil background images. (**ii**) Step 2: Deep learning models used for identifying corn and non-corn blocks within an image.

The developed framework for counting plants (Figure 3) is divided into two main steps: (i) the automatic annotation of an RGB image into smaller blocks of corn and non-corn using image enhancement techniques (discussed in Section 2.3.1) and (ii) the training and testing of four DL models including VGG16 [30], VGG19 [31], InceptionV3 [32], and Vision Transformer (ViT) [33] based on annotated image blocks to identify corn plants and then estimate the corn stand counts (discussed in Section 2.3.2). 

#### 2.3.1. Automatic Annotation of Images

To automate image annotation for developing DL models for corn counting, we first extracted corn row location information that was later used to create smaller annotated image blocks of corn and non-corn. To extract the crop rows’ location, an RGB orthomosaic image was rotated until the rows aligned with a perfectly straight horizontal orientation. The extent to which an image should be rotated was derived by estimating the crop row orientation in the center of the orthomosaic image. After rotation, horizontal strips of 1 m in width, matching the spacing between corn rows at the study site, were created. This ensured that each strip contained only one crop row, enabling the accurate extraction of row locations. A green area mask was then generated using upper and lower thresholds (36, 25, 25) and (70, 255, 255) on the RGB bands to identify corn plants. 

The green mask highlighted the green pixel positions that represented corn plant locations. Then, starting and ending corn pixel locations in each horizontal strip were utilized to fit a linear line and extract the crop row. Along the crop row, blocks of size 0.2 m × 0.25 m were created and checked with a 7% green area criterion to label them as corn plant-representing blocks. The image blocks of 0.2 × 0.25 m were represented by approximately 11 × 11 × 3 pixels, which were resized to match the required input size for each of the selected DL models using the cv2.INTER_AREA function (discussed in detail in Section 2.3.2). 

**Figure 4:**
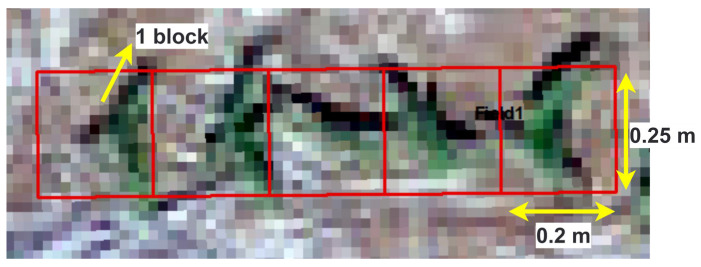
UAS image of sample area showing annotation block sizes of 0.2 m × 0.25 m.

This selected block size was found optimal for capturing corn plants at the V2–V4 growth stages (Figure 4). All the other blocks with less than 7% green area along the crop row and outside crop row were labeled as non-corn blocks. This labeling method ensured that areas outside the crop rows, where inter-row weeds might be present, were not mistakenly labeled as corn but as non-corn. The flowchart depicting this process is illustrated in Figure 3. These steps were implemented using functions from the OpenCV and Geopandas library in the Python platform.

#### 2.3.2. Deep Learning Models for Plant Counting and Their Architectures

*Training and testing datasets:* Given that our automatic annotation framework generates fixed-size image blocks annotated as corn or non-corn, we primarily focused on image classifier models such as VGG, Inceptionv3, and ViT rather than models that combine classification and detection algorithms like YOLO. YOLO requires bounding boxes around individual corn plants for training, whereas image classifier models can be trained on images without the need for such bounding boxes.

The annotated datasets were selected from various sections of the field, representing diverse field conditions, such as soil types, elevations, and soil backgrounds. This diverse selection ensures that each annotated image captures distinct color gradients, helping to build a robust generalized model capable of performing well across various test datasets. The selected training and validation datasets covered approximately 70% of the field, with the remaining 30% reserved for testing the DL models. Within this 70% dataset used for model training, a ratio of 70:30 was allocated for model training and validation, respectively. The dataset was further augmented by considering rotation, zoom, and horizontal flips using the ImageDataGenerator function in the Keras Python library for capturing variations in the annotated images. There were a total of 18,004 corn and 46,542 non-corn labeled images, which is a ratio greater than 2-to-1. 

*Approach to counter data imbalance for the training of DL models:* Studies have shown that highly imbalanced datasets tend to bias models toward the majority class—in our case, non-corn, resulting in a poor performance for the minority class, i.e., corn [34,35]. This occurs because the model has fewer features in the minority class to learn from compared to the majority. To improve classification results for the minority class, we addressed this imbalance by applying random undersampling, which reduced the number of images in the majority class in the training dataset. By selecting a random subset of images from the majority class, we ensured that both classes had an equal number of images for training the models. 

**Figure 5:**
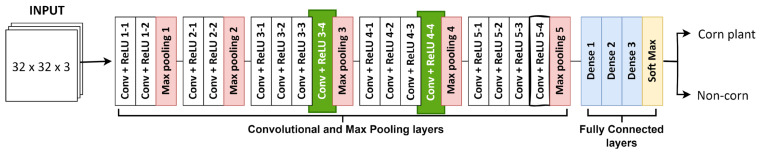
Architecture of VGG16 and VGG19 (with two extra convolutions and a ReLU layer marked in green) showing the connection of the convolutional, pooling, dense, and softmax layers.

*DL model architecture:* Three DL architectures were considered for corn stand counting as discussed below:

**VGG16**: It is an object recognition algorithm renowned for its simplicity and effectiveness. It employs multiple convolution layers with a 3 × 3 filter followed by a max-pooling layer with a 2 × 2 filter. At the end of the architecture, it has three fully connected layers followed by a softmax output. VGG19 has an architecture similar to that of VGG16, except for the addition of 3 convolutional layers added at stages 3, 4, and 5 (Figure 5). The input size for VGG16 and VGG19 is 32 × 32 × 3, representing images with a dimension of 32 × 32 and 3 color RGB bands.

**Figure 6:**
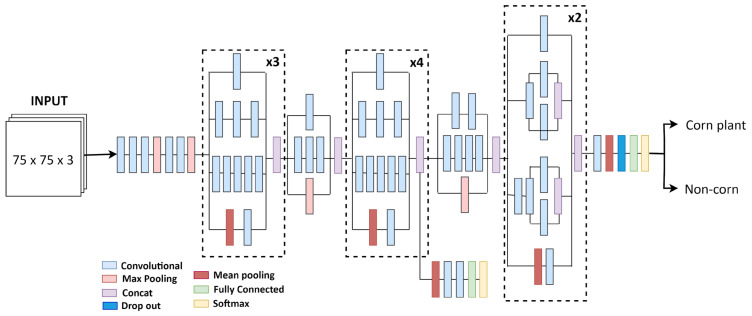
Architecture of InceptionV3 showing the connection of the convolutional, pooling, concat, and softmax layers.

**InceptionV3**: Unlike VGG16, InceptionV3 uses convolutions of varying sizes (1 × 1, 3 × 3, and 5 × 5) within the same module, serving as a multi-level feature extractor from input images, which are then aggregated along the channel dimensions (Figure 6). Additionally, InceptionV3 uses comparatively fewer parameters (weights and biases) than VGG models, which can make it more efficient in terms of compute time and resources. It requires a minimum input size of 75 × 75, so 75 × 75 × 3 was used for its training in this study. 

Table 1 outlines the input size, trainable parameters, and computational time for each model. VGG16 and VGG19 were configured with a few non-trainable layers, resulting in 7,116,546 training parameters, which were found sufficient for learning corn and non-corn images. In contrast, Inceptionv3 had all layers set as trainable, updating 21,772,450 parameters. Consequently, InceptionV3 exhibited a longer training time compared to VGG16 and VGG19 due to its higher parameter count. Inceptionv3 was trained with more parameters since fewer parameters resulted in poorer model performance. These models were trained with a batch size of 32 and achieved stable training accuracy around 30 epochs.

**Figure 7:**
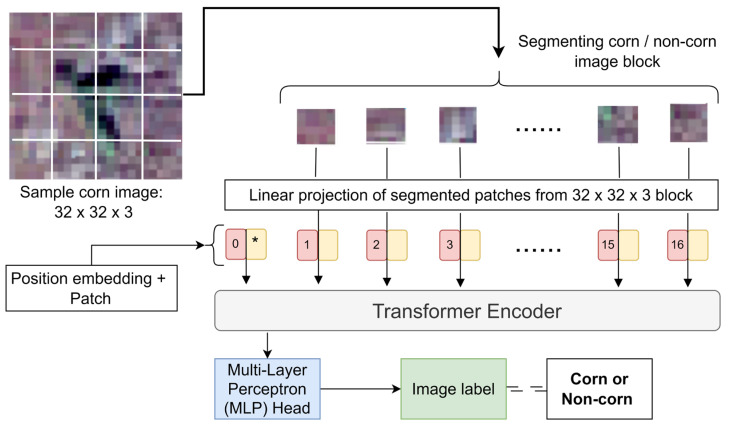
Architecture of ViT showing the position embedding on image patches before passing to the transformer encoder.

**ViT model:** In addition to the CNN-based models (Inceptionv3, VGG16, and VGG19), we developed a transformer-based ViT model, which uses an attention mechanism in its encoder unit of the transformer architecture. The model processes annotated images by dividing them into user-defined image patches, which are linearly embedded into one-dimensional vectors. These vectors, combined with image positional information, are passed into the transformer encoder. The encoder’s self-attention mechanism estimates the importance of each embedded image patch, enabling the model to learn long-range dependencies between patches (Figure 7). Despite having fewer training parameters than other models, the ViT model did not achieve significant performance gains with additional parameters. An 8 × 8 image patch size was chosen, as it outperformed 4 × 4 patches. The ViT model achieved stable training accuracy after 30 epochs with a batch size of 8. 

These models were built in a machine with a configuration of 64 GB of RAM and an Intel(R) Xeon(R) Silver 4114 CPU @ 2.20 GHz processor. The keras and scikit-learn Python libraries were used to implement these DL models.

*Evaluating DL model generalizability for corn stand counting using random seeds:* In any DL and ML model, a random seed is used to initialize the model, leading to different starting states and the random selection of training and testing images. This can lead to varying performances of the same model with different datasets. To assess the generalizability of each DL model for corn stand counting, we trained five versions of each DL model, using different training and testing datasets, each selected based on a unique random seed. The models were then compared in terms of loss, accuracy, R^2^, and RMSE (see Section 2.4). In total, 20 models (five versions for each of the four DL models) were evaluated using different testing datasets to ensure an unbiased assessment of the models. 

**Figure 8:**
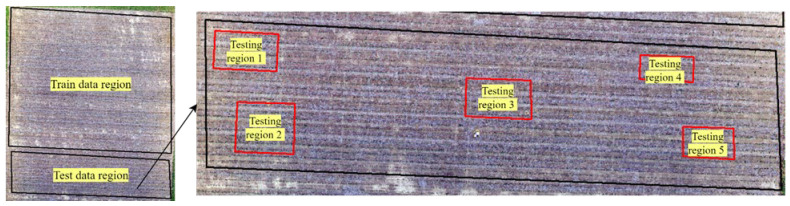
Five test regions were selected to compare the manually counted plant stand counts with the model results.

After training the models, their performance was evaluated using test data from five randomly selected regions within the field (Figure 8), ensuring that these regions were distinct from those used in training for an unbiased assessment. Following the model’s prediction, a post-processing step was used, which involved filtering out corn plant locations with model probabilities below 90%, effectively removing low-confidence results. 

*Post-processing of DL-based outputs to prevent double counting*: When the orthomosaic image was automatically divided into smaller image blocks for the training and testing of the models, some blocks contained different portions of the same plant. To prevent the double counting of the same corn plant across multiple images, adjacent image blocks representing the same corn plant were removed using the Intersection over Union (IoU) method. This method eliminates overlapping bounding boxes based on their intersection area. After this post-processing step, corn plants were counted in the five test regions and compared with the manual observations.

### 2.4. Model Performance Evaluation

**Figure 9:**
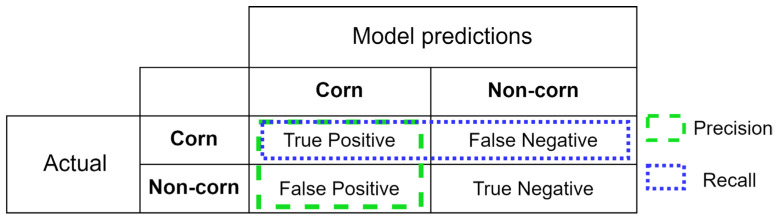
A confusion matrix for corn plant stands. Positive refers to corn plant stand and negative refers to non-corn.

The performance of DL models was assessed using precision, recall, and F1 score metrics, which were calculated by comparing the model’s corn stand estimations with manually labeled corn plant stands (Equations (1)–(3)). Higher precision, recall, and F1 score correspond to higher model accuracy. In these equations, True Positive (TP) represents the model’s ability to correctly identify corn plants within the ground reference corn blocks, while False Positive (FP) refers to instances where the model incorrectly predicts a corn plant when it is not actually present. False Negative (FN) indicates the model’s failure to detect an actual corn plant. A confusion matrix showing the TP, FP, TN, and FN are explained in Figure 9.

(1) $$\mathrm{Precision}=\frac{\mathrm{True}\;\mathrm{Positive}}{\mathrm{True}\;\mathrm{Positive}+\mathrm{False}\;\mathrm{Positive}}$$

(2) $$\mathrm{Recall}=\frac{\mathrm{True}\;\mathrm{Positive}}{\mathrm{True}\;\mathrm{Positive}+\mathrm{False}\;\mathrm{Negative}}$$

(3) $$F1=2\times (\frac{\mathrm{Precision}\times \mathrm{Recall}}{\mathrm{Precision}+\mathrm{Recall}})$$

After predicting corn stand counts using the model and performing post-processing, the results were counted in the five test regions and compared with manually observed corn stands (referred to as the ground truth) to estimate the coefficient of determination (R^2^) and Root Mean Square Error (RMSE) (Equations (4) and (5)):

(4) $$R^{2}=1-\frac{\sum_{1}^{n}{\left(x_{i}-p_{i}\right)}^{2}}{\sum_{1}^{n}{\left(x_{i}-x_{m}\right)}^{2}}$$

(5) $$\mathrm{RMSE}=\sqrt{\frac{\sum_{1}^{n}{\left(x_{i}-\;p_{i}\right)}^{2}}{n}}$$

where $x_{i}$ represents the manual observation of the corn stand count in the ith sample test region, $p_{i}$ represents the corn stand count estimated from the DL model at the ith sample test region, $x_{m}$ denotes the mean stand count of manual observations, and n represents the number of sample test regions. Higher R^2^ and lower RMSE values correspond to a better performance of the DL model. By comparing these values across the four different DL models, we can assess the models’ effectiveness in identifying corn stands in the UAS images of the field.

## 3. Results

### 3.1. Comparative Analysis of Automated and Manual Approaches in Curating Data for the Training and Test Set

**Figure 10:**
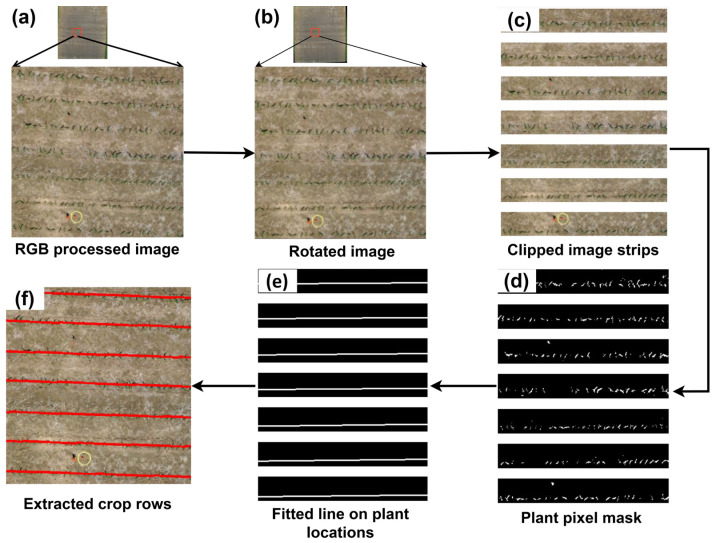
Sequence of steps considered in automatically generating corn rows from a field image: (**a**) RGB orthomosaic map representing a small section of the corn field, (**b**) rotated RGB image with rows laid horizontal after finding a rotating angle based on the image orientation, (**c**) image strips considering a 1 m inter-crop row spacing on the rotated image, (**d**) mask representing green pixels in each image strip based on upper and lower thresholds of green pixel values, (**e**) fitted linear lines representing corn rows based on the locations of the green masks, and (**f**) identified corn rows overlaid on RGB image.

The creation of annotated corn and non-corn image blocks for training DL models relied on the extraction of corn rows from the orthomosaic RGB image. The results of this process are shown in Figure 10. These extracted crop rows were examined and overlaid onto the corresponding UAS image using the ArcGIS software (10.7). This evaluation was conducted to assess the effectiveness of the crop row extraction method, which demonstrated an accuracy of 85–90% in representing crop rows compared to UAS images. The method successfully captured most corn rows, particularly when weeds were absent between corn rows. The accuracy of the method, however, declined in the presence of inter-row weeds due to the similarity in color gradients between weeds and corn plants.

**Figure 11:**
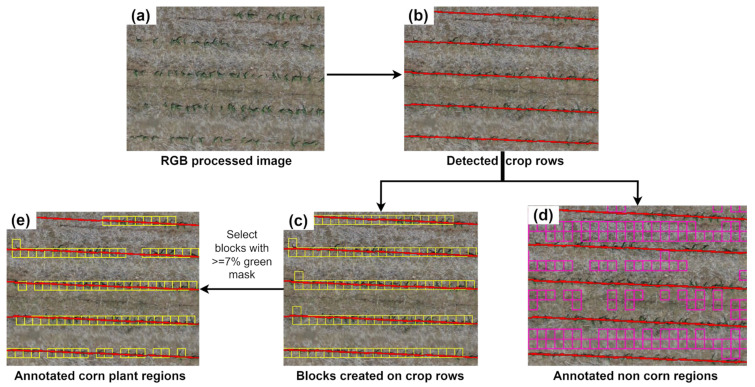
Annotated image blocks of corn and non-corn stands in the field. (**a**) RGB image of a small section of the study site, (**b**) corn rows detected using the crop row detection method, (**c**) extracted corn plant blocks intersecting with corn rows, (**d**) extracted non-corn blocks, and (**e**) filtered corn blocks using the percentage of green pixels.

Following the extraction of corn rows, image blocks of 0.2 m × 0.25 m size were created and aligned along the corn rows. The blocks containing a minimum of 7% green pixels of the total image area were then annotated as corn plant blocks (Figure 11). Blocks within the row but with less than 7% of green pixels, along with those outside the crop rows, were annotated as non-corn blocks. This process generated annotated training data of two categories, corn and non-corn, across various soil backgrounds within the field. The accuracy of annotated image blocks was assessed by aligning them with the RGB orthomosaic, mirroring a manual approach, at approximately 20 randomly selected locations. This analysis demonstrated that the proposed method accurately captured corn and non-corn image blocks, identifying 80% of total corn plants. While this approach effectively represented corn plants with a decent accuracy, the annotated images were further used to train four DL models to further enhance the classification accuracy of corn stands.

### 3.2. Performance of Deep Learning Models for the Classification of Corn Plants

**Figure 12:**
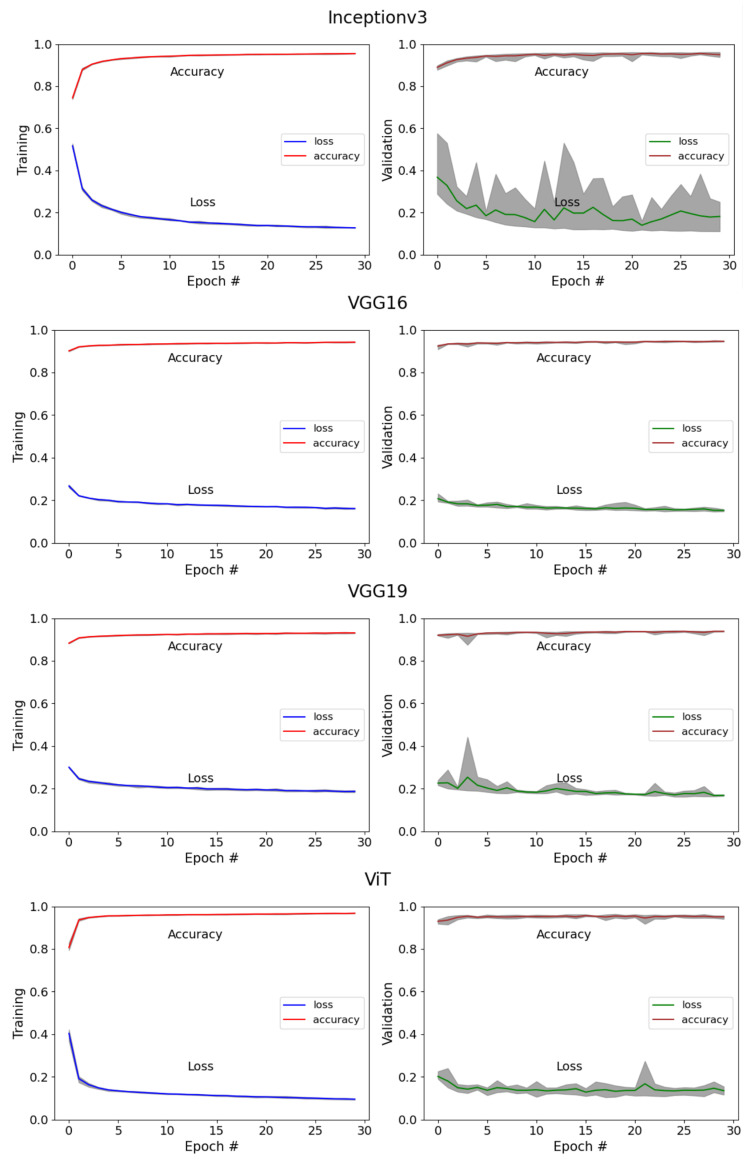
Distribution of loss and accuracy for four DL models over 0 to 30 epochs during training and validation phases. In each graph, the solid lines represent the average loss and accuracy across five versions of the same DL model, with each version trained and validated on different datasets. The gray-shaded areas around the lines indicate the range, showing the maximum and minimum loss and accuracy values across the five versions at each epoch. # refers to the epoch number.

In building on the evaluation of the generalizability of the DL models for corn stand counting, the training and validation accuracy and loss for the four models across the five randomly selected datasets are shown in Figure 12. The figure highlights the central trends and the variability in performance across different datasets. At epoch 30, the final training accuracy was 0.88, 0.92, 0.91, and 0.93 for Inceptionv3, VGG16, VGG19, and ViT, respectively. Throughout the training phase, all four models exhibited minimal variation in loss and accuracy across all datasets.

During the validation phase, VGG16, VGG19, and ViT showed stable accuracy and loss values across the datasets, with the exception of InceptionV3, which displayed more variability. This suggests that the random sampling of training and validation data can significantly influence model learning, particularly for Inceptionv3. Some of this could be attributed to its model architecture involving varying filter sizes and convolution layers [35]. In contrast, VGG16, VGG19, and ViT consistently exhibited stability across four of the five datasets, showcasing their robust performance. 

Although Inceptionv3 demonstrated higher accuracy when trained with more epochs (50, 100, and 150), its validation accuracy was still unstable, with accuracy ranging between 0.5 and 0.94. In contrast, ViT, VGG16, and VGG19 demonstrated robust performance across different random datasets, with VGG16 consistently outperforming VGG19 and ViT in both training and validation. This stability, combined with lower computational requirements, led to halting the training of Inceptionv3 with additional epochs. Furthermore, increasing the number of epochs and hyperparameter tuning had minimal impact on model performance. 

**Figure 13:**
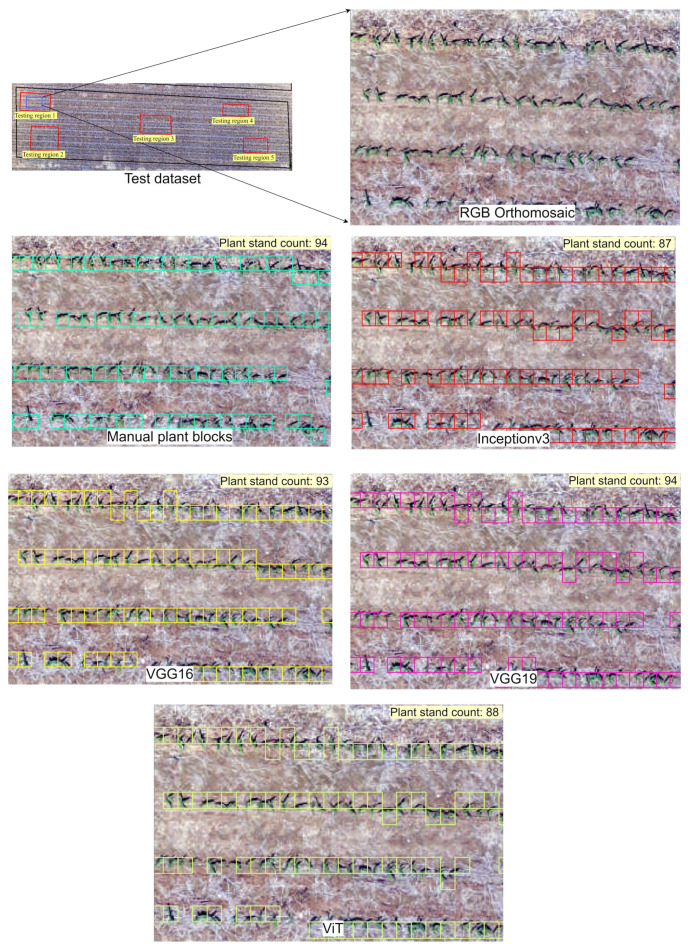
The models’ performance in detecting corn plant blocks within a sample test region. The colored blocks overlaid on the UAS images represent the corn plant blocks identified by the DL models. Each colored block represents the predicted corn plant blocks identified by a different model.

To further assess the performance of DL models, the trained models were tested on randomly selected regions within the field. Each test image block, which represents a portion of the field, was processed by the trained models to identify blocks containing corn plants (Figure 13). Although these blocks were non-overlapping and might cover only a part of the corn stands, the models were still able to predict the presence of corn plants. Post-processing was applied to remove duplicate predictions based on adjacent overlap information, and the final corn plant blocks were compared with manual visual observations to evaluate the accuracy of the models’ predictions. 

**Figure 14:**
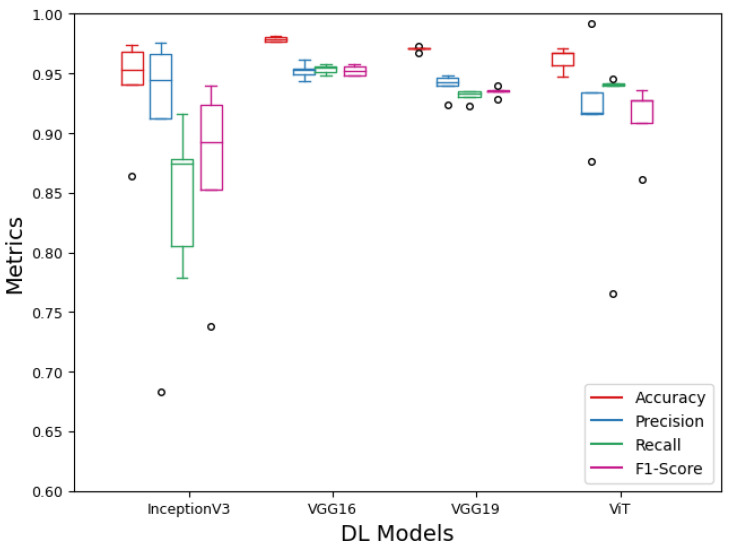
Comparison of accuracy, precision, recall, and F1 score of Inceptionv3, VGG16, VGG19, and ViT models on the five random test regions in the corn field. The small black circles represent the outliers, indicating the extremely low and high values observed across five versions of each model.

The model performance was further evaluated using metrics, such as accuracy, precision, recall, and F1 score, as depicted in Figure 14. Comparative analyses of these metrics revealed that VGG16 and VGG19 outperformed InceptionV3 and ViT. VGG16, in particular, achieved the highest F1 score of 0.955 for the dataset generated with random seed five. Although all models demonstrated good precision, indicating high accuracy in positive predictions, InceptionV3 had lower recall values, suggesting a higher number of false negatives. In contrast, ViT consistently had high recall. The F1 scores further highlighted the stable performance of VGG16 and VGG19, with the highest values being 0.955 and 0.939, respectively. ViT also performed well, achieving an F1 score of 0.935, and showing stability across various dataset except the fourth. Among the four models, VGG16 exhibited consistently stable and high scores across all performance metrics. 

### 3.3. Model Predicted vs. Manually Counted Corn Stands

**Figure 15:**
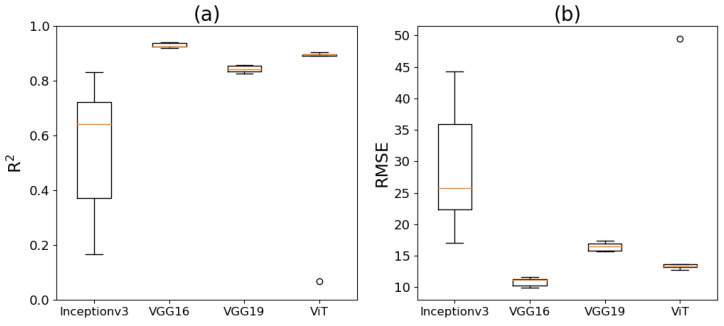
Performance of Inceptionv3, VGG16, VGG19, and ViT models based on R^2^ (**a**) and RMSE (**b**) on five random test regions in the corn field. The orange line in the boxplot represents the median model performance value.

The models’ estimated corn plant stands and the manually counted corn plant stands in the five test regions are shown in Table 2. The model performance was evaluated using the R^2^ and RMSE, with variations across different datasets illustrated in Figure 15. Detailed R^2^ and RMSE values for all versions of DL models can be found in Table S1. Notably, Inceptionv3 exhibited a superior performance with the second dataset (i.e., random seed two), but its R^2^ and RMSE values varied widely with other datasets, reflecting poor consistency in detecting corn blocks. In contrast, VGG16, VGG19, and ViT (except for only one out of five datasets) outperformed Inceptionv3, with higher R^2^ and lower RMSE values. VGG16 and VGG19 consistently exhibited a narrow range of R^2^ and RMSE values across five datasets. In particular, VGG16, trained with the second dataset achieved a high R^2^ of 0.94 and a lower RMSE of 9.95. On average, Inceptionv3, VGG16, VGG19, and ViT had mean R^2^ values of 0.55, 0.93, 0.84, and 0.73, respectively, with corresponding mean RMSE values of 29, 10.86, 16.45, and 20.48. Overall, VGG16 demonstrated high accuracy with minimal variation in model performance across different random datasets, while ViT showed stable performance except for one dataset. For detailed scatterplots comparing model-predicted corn stand counts with the manual counts across all model versions, please refer to Figure S1 in the Supplementary Materials.

## 4. Discussion

### 4.1. Automating High-Quality Annotated Training Data for Corn Counting

The effectiveness of DL models in crop identification hinges largely on the quality and quantity of the training data. Previous studies have often developed DL models using manually annotated images, which can be labor intensive. For instance, Wu et al. (2019) [28] estimated a resource requirement of approximately 123 person-hours for annotating images to develop a rice seedling count model. In their study, rice seedlings were manually labeled as points (instead of blocks) in 40 high-resolution images, with seedlings counts varying from 3732 to 16,173. In a separate study [17], researchers manually labeled only 864 images using Labelme software for training and testing the YOLOv3 model in detecting corn seedlings. This limited dataset poses a potential challenge, as the implemented model may underperform in scenarios where the data lack diversity. 

This study explored automating the generation of high-quality annotated training datasets by leveraging crop row information extracted through image morphological approaches [27,36,37], with the goal of developing DL models trained on these annotated images for accurate corn counting. Unlike previous studies that relied on limited training datasets, our study introduced a workflow that automatically generates 18,004 corn images and 46,542 non-corn images for a 1.5-acre corn field, which were then used to train the corn counting model. Once the annotation method was implemented, extracting and annotating these image blocks took 4–5 h, including the time to generate orthomosaic image, identify crop rows, and refine the labeled images. The primary benefit of this process is its future scalability; the framework can be applied to new fields to quickly generate annotated datasets. With minor adjustments, we believe that this approach can help significantly streamline the process of generating annotated images and improve crop count estimates, thereby reducing significant time and labor costs. 

### 4.2. Deep Learning Models for Counting Plant Stands

In this study, DL models trained on automatically annotated images achieved an F1 score of up to 0.955, an R^2^ of up to 0.94, and an RMSE as low as 9.95 in detecting corn plant stands. The performance of these DL models was comparable to the results of previous studies based on manually annotated images [17,38,39]. For example, a recent study [39] using YOLOv5, YOLOv7, and CenterNet models trained on manually annotated images (using LabelImg tool) achieved an F1 score between 0.90 and 0.95 for detecting cotton seedlings. This highlights the efficacy of our approach in generating annotated images for training DL models. 

### 4.3. Differences in Performances with Other Models 

In this study, VGG16 demonstrated the best performance in corn counting, followed by ViT, VGG19, and InceptionV3. However, it is essential to note that ViT was trained with the fewest number of parameters compared to the rest of the models. Hence, if time is a big constraint in building a DL model for corn counting, ViT can be a viable alternative without sacrificing much in performance. It is also worth pointing out that there are other DL architectures, such as YOLO, which could provide a very precise estimate of plant bounding boxes compared to our model-predicted corn plant blocks. Future studies could explore these architectures for further improvement.

Apart from DL analyses, object-based image analysis (OBIA) has also been used to count crop plants. Koh et al. (2019) [40] employed template matching for detecting safflower seedlings at early growth stages, achieving an R^2^ of ~0.87 and an RSME of ~10. Template matching is an image processing technique wherein a template image is utilized to match smaller segments within a larger image. This study acquired data at a resolution of 0.19 cm/pixel and automated the OBIA algorithm using the proprietary eCognition (9.3) software. The results obtained in our study surpassed these metrics using an open-source Python platform, offering ease of development and deployment to diverse row crop fields.

## 5. Limitations and Future Works

In this study, the automated image annotation framework was developed and tested for corn stand counting. However, the developed framework can easily be adapted to other crops with minimal parameter adjustments, such as crop row orientation, typical crop coverage in a given growth stage to determine the size of image blocks, and the percent threshold of green pixels within a crop block, to improve labeling. It is also important to note that the image annotation framework assumes that the crop rows are straight rather than curved, as corn fields in the U.S. are typically planted using GPS-based guidance systems [41], which helps maintain straight lines, improve planting accuracy, minimize crop overlap, and eventually ensure consistent application of inputs and fertilizers throughout the growing season. The field used in this study is representative of many U.S. corn fields and features predominantly straight rows, with the exception of the edges, which are primarily used for turning agricultural machinery. Hence, we used a linear method for row detection. However, this approach may struggle to identify curved rows, potentially reducing the accuracy of corn counting models trained on data annotated using curved rows’ information. Future work could focus on developing methods capable of detecting curved rows, thus enhancing the robustness of crop counting models across various planting scenarios.

Similarly, the effectiveness of our approach in corn row identification and stand counts may be affected by factors such as the presence of doubles and seed spacing accuracy. Our row crop identification method relies on consistent inter-row spacing and performs best when doubles are absent, and when spacing between corn stands is uniform. While we acknowledge that these issues can influence the accuracy of our method, most row crop fields in the U.S. are planted using modern precision technology designed to minimize doubles through more accurate seed placement. The corn field used for our study was planted using an RTK GPS-based precision planter, and there was no obvious presence of doubles. Nevertheless, future research could focus on developing corn counting models that maintain high accuracy even in the presence of doubles and inconsistent seed spacing. 

Here, our DL models for corn stand counting were trained exclusively on images from a single corn field in V2–V4 growth stages. The robustness of these models can be improved by incorporating images from a range of field sites, environmental conditions, crop growth stages, and management practices. This helps to build a larger, diverse database, which will ultimately enhance model performance and the transferability of a model to new fields. 

## 6. Conclusions

In this study, we developed an automated image annotation framework that utilizes image enhancement techniques to annotate the images, which were used for the training of four DL models, including InceptionV3, VGG16, VGG19, and ViT for the accurate detection and counting of corn stands. While Inceptionv3 exhibited relatively unstable performance and its performance was sensitive to the random selection of training and validation datasets, VGG16, VGG19, and ViT demonstrated more stable performance, indicating their better adaptability to varying training datasets. Notably, VGG16 outperformed the other three models, achieving an F1 score of 0.955, an R^2^ of 0.94, and an RMSE of 9.95 when compared to corn stand counts in the test regions. ViT provided the second-best performance, with an R^2^ of 0.90 across four out of five training datasets. Overall, the developed automated labeling framework for training the DL model, especially the VGG16 model, demonstrated promising potential for accurate and efficient corn stand detection. This approach paves the way for automating the generation of training data, contributing to the development of a more robust and effective corn plant identification model.

## Figures and Tables

**Table 1:** Model specifications and computational time.

Model	Image Input Size	Trained Parameters	Training Time (Mins)
VGG16	32 × 32 × 3	7,116,546	300
VGG19	32 × 32 × 3	7,116,546	320
InceptionV3	75 × 75 × 3	21,772,450	750
ViT	32 × 32 × 3	275,394	200

**Table 2:** Total corn stand counts at five test regions (TRs) after the training of four deep learning models.

		TR1	TR2	TR3	TR4	TR5
Ground Truth		229	274	184	116	140
Models	Version		Estimated Corn Stand Counts			
**Inceptionv3**	1	205	246	162	99	121
	2	198	236	176	100	116
	3	203	255	170	103	133
	4	194	203	155	92	94
	5	192	233	150	96	97
**VGG16**	1	209	263	177	111	135
	2	211	263	177	111	137
	3	210	259	181	111	136
	4	209	261	177	110	135
	5	210	266	178	111	137
**VGG19**	1	202	255	177	111	132
	2	200	252	178	109	135
	3	203	251	177	107	135
	4	207	249	176	109	135
	5	202	251	173	108	132
**ViT**	1	206	259	180	108	133
	2	210	255	179	110	134
	3	204	260	179	108	135
	4	146	235	133	87	120
	5	206	260	180	107	133

Note: There were five versions for each DL model, each trained and validated with different training and validation datasets, selected based on five unique seeds.

## Data Availability

Data used in this study can be made available upon request.

## Supplementary Materials

The following Supporting Information can be downloaded at https://www.mdpi.com/article/10.3390/s24196467/s1: Table S1: R2 and RMSE values observed for all DL model versions; Figure S1: Comparison of corn stand count with the manual count for five test regions observed for each DL model.
