# Supplementary material for: Integrating Automated Labeling Framework for Enhancing Deep Learning Models to Count Corn Plants Using UAS Imagery
Source: Sensors (Basel). 2024 Oct 7;24(19):6467. doi: 10.3390/s24196467 (PMC11479280; doi:10.3390/s24196467)
Supplement: Supplementary file 1 [file sensors-24-06467-s001.zip › sensors-3163606-supplementary.pdf]

Supporting Document

Integrating Automated Labeling Framework for Enhancing Deep Learning Models to Count Corn Plants Using UAS Imagery

Sushma Katari [1,*], Sandeep Venkatesh [2], Christopher Stewart [3] and Sami Khanal [1,*]

[1] Department of Food, Agricultural, and Biological Engineering, Ohio State University, 590 Woody Hayes Dr, Columbus, OH 43210, USA
[2] Google, Kirkland, WA 98033, USA
[3] Department of Computer Science and Engineering, Ohio State University, 590 Woody Hayes Dr, Columbus, OH 43210, USA
* Correspondence: katari.5@osu.edu (S.K.); khanal.3@osu.edu (S.K.)

Table S1: R^2 and RMSE values observed for all DL model versions.

Model	Versions	R^2	Mean R^2	RMSE	Mean RMSE
**InceptionV3**	1	0.72	**0.55**	22.33	**29.052**
	2	0.64		25.69	
	3	**0.83**		**17.04**	
	4	0.17		44.27	
	5	0.37		35.93	
**VGG16**	1	0.93	**0.93**	11.14	**10.86**
	2	**0.94**		10.28	
	3	0.92		11.28	
	4	0.92		11.65	
	5	**0.94**		**9.95**	
**VGG19**	1	**0.86**	**0.844**	**15.67**	**16.45**
	2	0.83		16.94	
	3	0.84		16.49	
	4	**0.86**		15.79	
	5	0.83		17.36	
**ViT**	1	**0.90**	**0.73**	13.29	20.43
	2	0.90		**12.8**	
	3	0.89		13.67	
	4	**0.07**		49.50	
	5	0.90		13.20	

Note: Each DL model had five versions, each trained and validated with different training and validation datasets, selected based on five unique seeds.

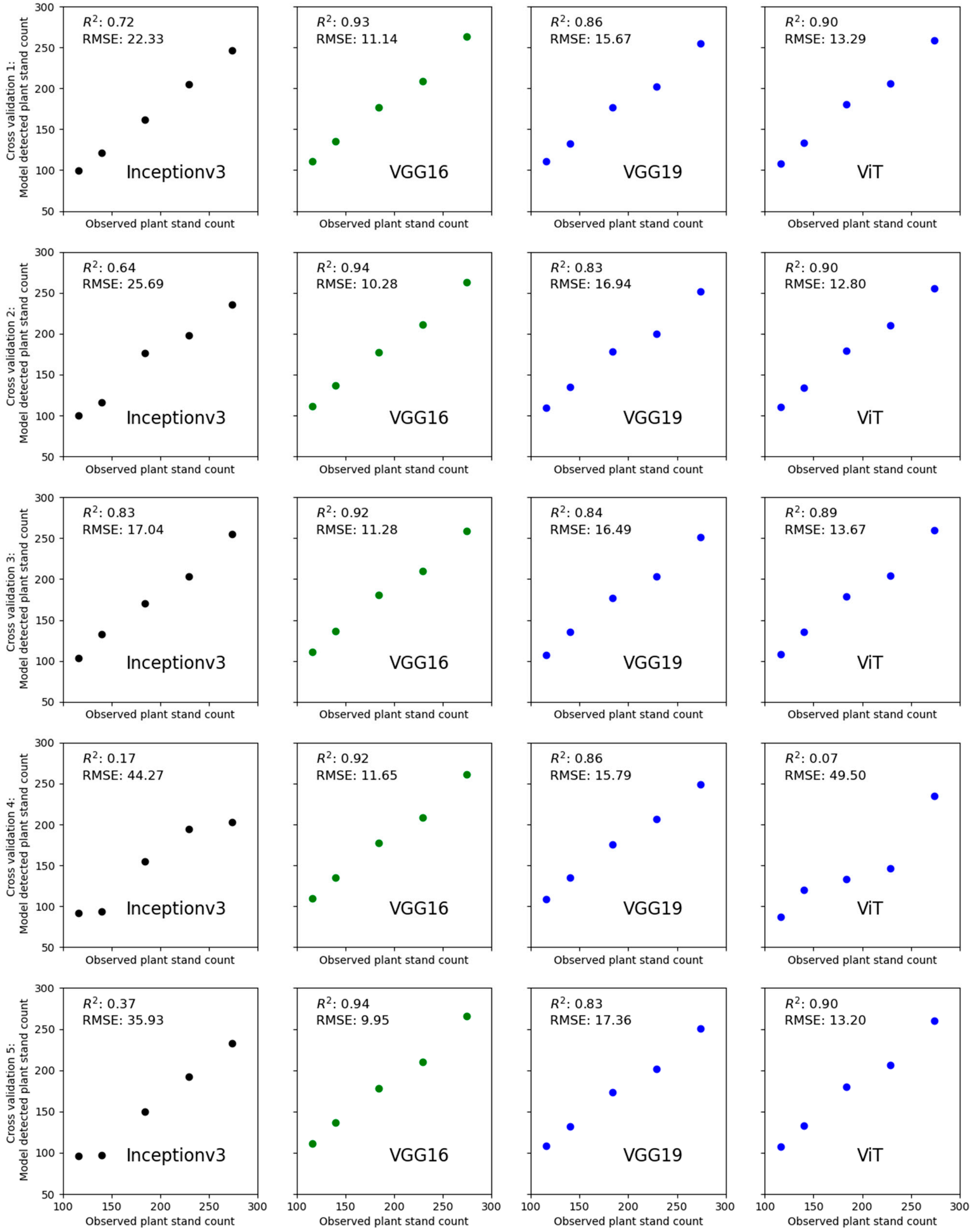

Figure S1: The comparison of corn stand count with the manual count for five test regions observed for each DL model.
